# Supplementary material for: Arbitrary waveform AC line filtering applicable to hundreds of volts based on aqueous electrochemical capacitors
Source: Nat Commun. 2019 Jun 28;10:2855. doi: 10.1038/s41467-019-10886-7 (PMC6598994; doi:10.1038/s41467-019-10886-7)
Supplement: Supplementary file 1 — Supporting Information [file 41467_2019_10886_MOESM1_ESM.pdf]

Supplementary Information

Arbitrary Waveform Applicable AC Line Filtering to Hundreds of Volts Based on Aqueous Electrochemical Capacitors

Wu et al.

Supplementary Results and Discussion

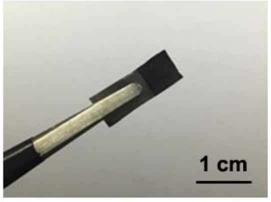

Supplementary Figure 1. Optical image of the PEDOT positive electrode. The black square is PEDOT on the grey graphite foil.

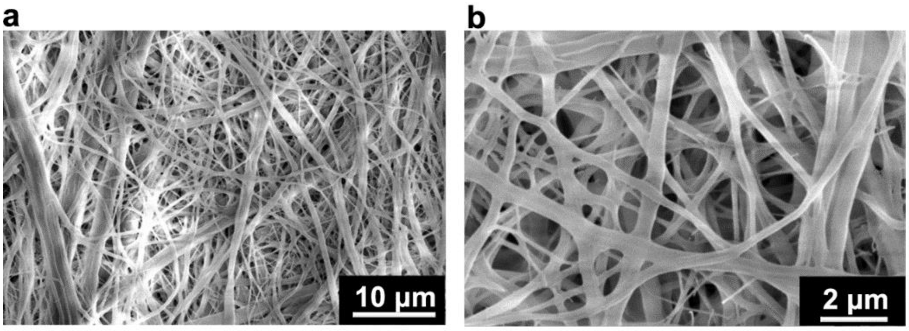

Supplementary Figure 2. SEM images of cellulose membrane with the fibril structure.

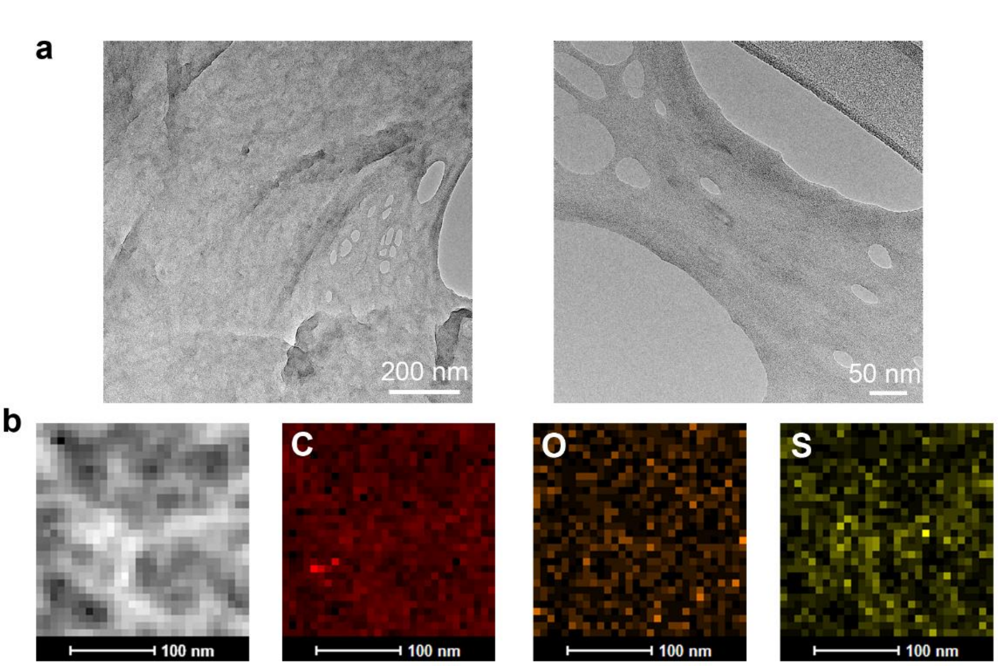

Supplementary Figure 3. Structure characterization of PEDOT. **a** TEM images of PEDOT positive electrode. **b** EDS mappings of C, O and S element for PEDOT positive electrode.

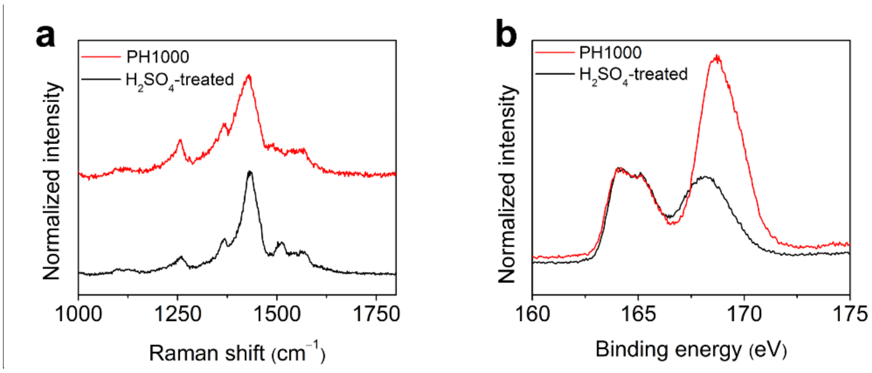

Supplementary Figure 4. Chemical Structures of PEDOT before and after H_2SO_4 treatment. **a** Raman and **b** S 2p XPS spectra of PEDOT positive electrode before and after H_2SO_4 treatment. Source data are provided as a Source Data file.

The pristine PH1000 is composed of PEDOT part and poly-(4-styrenesulfonate) (PSS) part which act as the dopant and dispersant of PEDOT chain[1]. Acid treatment would induce the protonation of PSS chain and weaken the interaction between the PEDOT and PSS, leading to the releasing of PSS, which could be seen from the Raman spectra and the XPS analysis (Supplementary Figure S4)[2–5]. In the Raman spectra, the 1430 cm^{-1} band and 1510 cm^{-1} are assigned to the symmetric and anti-symmetric $C_\alpha=C_\beta$ stretching vibrations of thiophene rings. The sharper band at 1510 cm^{-1} represent the longer conjugation chains after acid treatment. In addition, the S 2p XPS spectra reveal two typical peaks, corresponding to the S atoms of PEDOT chain at 164.4 eV band and the S atoms of PSS chain at 168.6 eV band. After the treatment of concentrated H_2SO_4, the obviously reduced intensity of 168.6 eV band indicate partial PSS chain was removed. Thus, acid-treated PEDOT positive electrode has an enhanced conductivity.

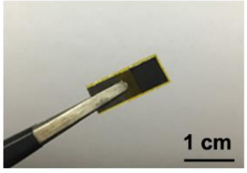

Supplementary Figure 5. Optical images of the ErGO negative electrode**.**

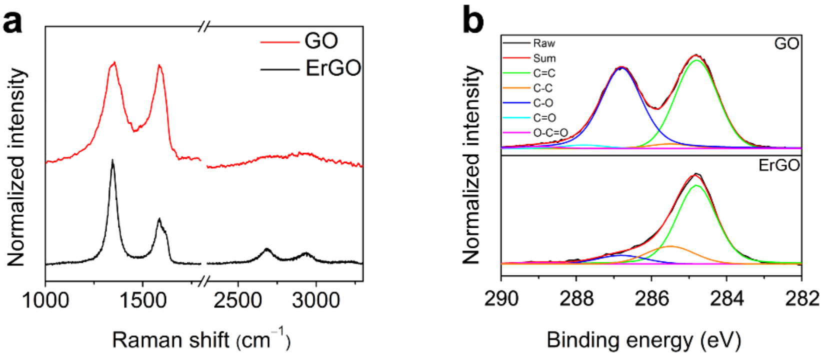

Supplementary Figure 6. Chemical structures of GO and ErGO. **a** Raman and **b** C 1s XPS spectra of freeze-dried ErGO negative electrode. Source data are provided as a Source Data file.

Typical Raman spectra of GO and ErGO involves the 1330-1340 cm^{-1} (D), 1580-1600 cm^{-1} (G), and 2670-2720 cm^{-1} (2D) band of carbon[6]. The average distance between defects (L_D) on graphene sheets could be calculated by using the intensity ratio of I_D to I_G (I_D/I_G). After electrochemical reduction, the ErGO shows an increasing L_Ds of 1.84 nm compared with the 1.01 nm of GO, indicating the well reduction of the ErGO sheets. In addition, the XPS spectra also prove this. Upon electrochemical reduction, the main peak of the oxygenated carbon atoms (C−O, C=O, and O−C=O) show a significant intensity decreasing, reflecting the remove of oxygenated group on the graphene sheets.

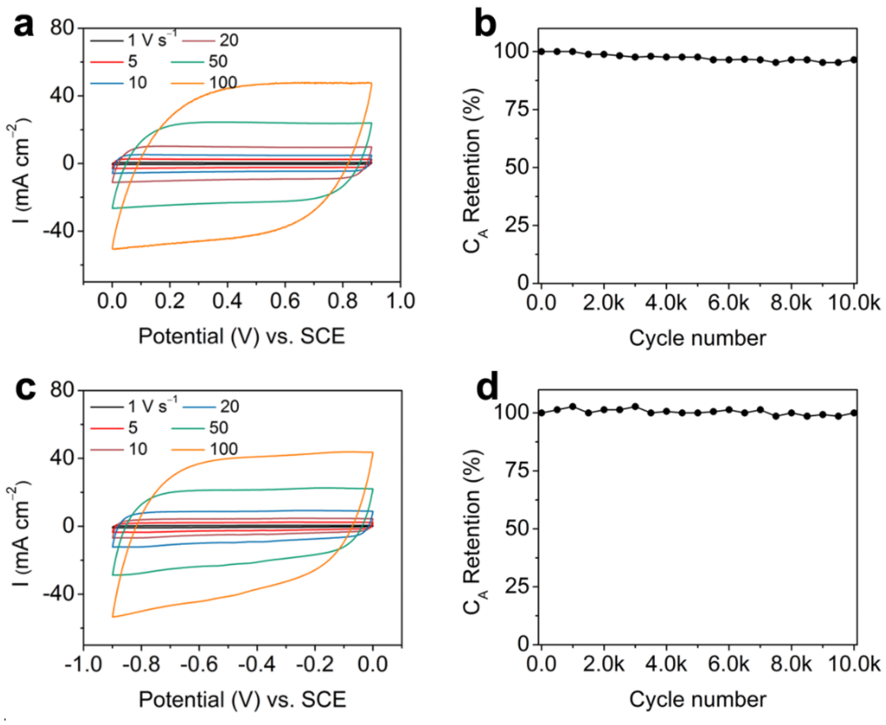

Supplementary Figure 7. Electrochemical performances of PEDOT and ErGO in three electrode system at 0-0.9 V vs SCE and −0.9-0 V vs SCE, respectively. **a** CV curves of PEDOT positive electrode within voltage window of 0-0.9 V vs SCE at different scan rates. **b** Cycling test of PEDOT positive electrode at 5 mA cm^{-2} within the voltage window of 0-0.9 V vs SCE for 10,000 cycles. **c** CV curves of ErGO negative electrode within voltage window of −0.9-0 V vs SCE at different scan rates. **d** Cycling test of ErGO negative electrode at 5 mA cm^{-2} within the voltage window of −0.9 of 0 V vs SCE for 10,000 cycles. Source data are provided as a Source Data file.

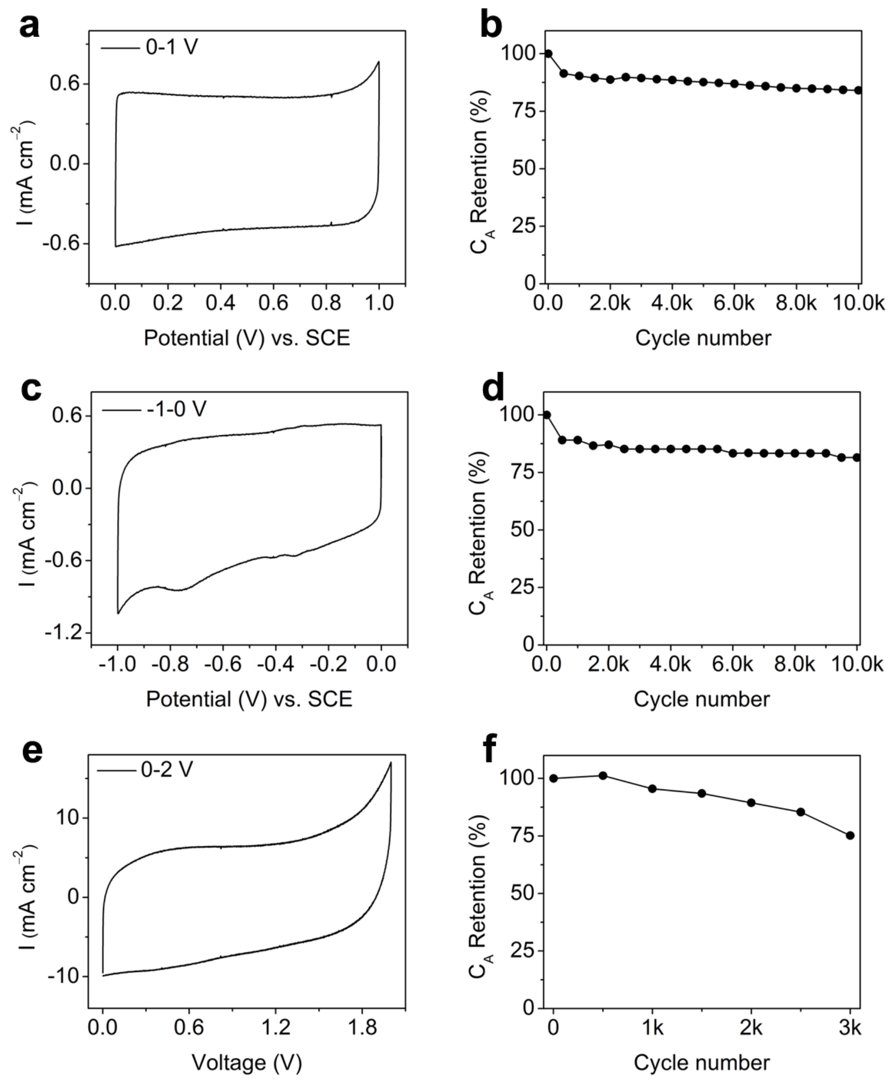

Supplementary Figure 8. Electrochemical performances of PEDOT, ErGO, and AHEC within 0-1 V vs SCE, −1-0 V vs SCE, and 0-2 V, respectively. **a** CV curve of PEDOT positive electrode within the voltage window of 0-1 V vs SCE (scan rate: 1 V s^{-1}). **b** Cycling stability test of PEDOT positive electrode within voltage window of 0-1 V vs SCE at 5 mA cm^{-2} for 10,000 cycles. **c** CV curve of ErGO negative electrode within the voltage window of −1-0 V vs SCE (scan rate: 1 V s^{-1}). **d** Cycling stability test of PEDOT positive electrode within voltage window of −1-0 V vs SCE at 5 mA cm^{-2} for 10,000 cycles. **e** CV curve of AHEC unit within the voltage window of 0-2 V (scan rate: 10 V s^{-1}). **f** Cycling stability test of AHEC unit within voltage window of 0-2 V at 5 mA cm^{-2} for 3,000 cycles. Source data are provided as a Source Data file.

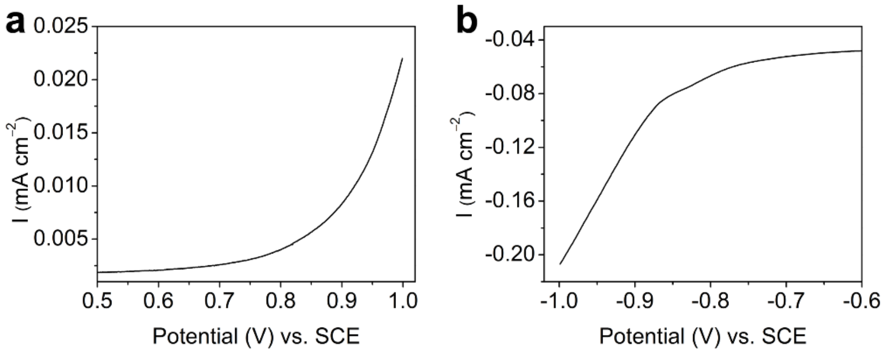

Supplementary Figure 9. Linear sweep voltammetry (LSV) curves of the positive electrode and negative electrode. **a** positive electrode and **b** negative electrode in 1 M Na_2SO_4 with the potential sweep at 1 mV s^{-1}, demonstrating the low activities of oxygen evolution reaction (OER) at 0.9 V vs SCE for the PEDOT and hydrogen evolution reaction (HER) at −0.9 V vs SCE for ErGO. Source data are provided as a Source Data file.

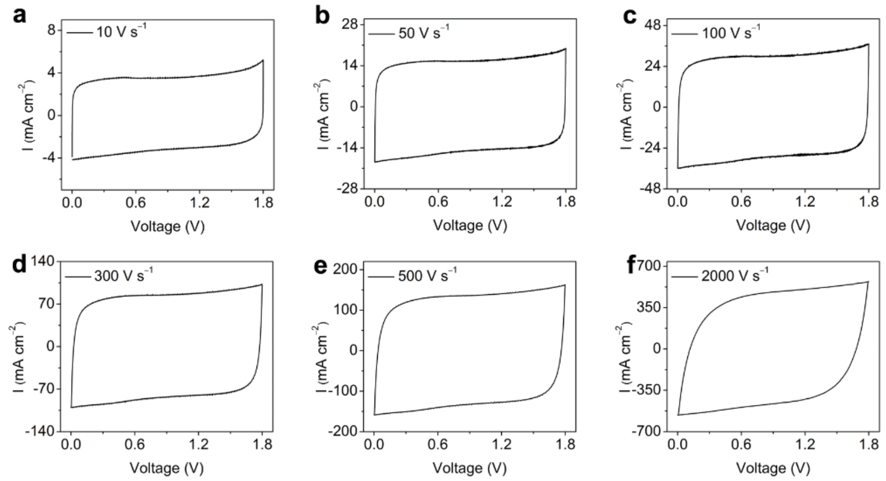

Supplementary Figure 10. CV curves of the AHEC at different scan rates. Source data are provided as a Source Data file.

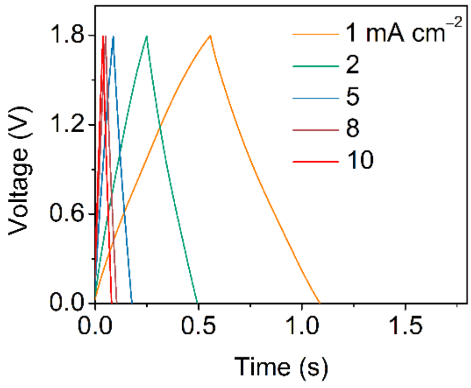

Supplementary Figure 11. GCD curves of AHEC at different current densities. Source data are provided as a Source Data file.

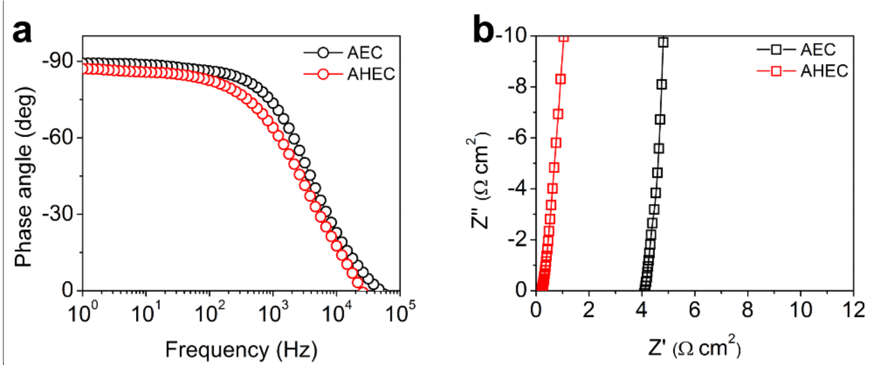

Supplementary Figure 12. Comparison electrochemical performances of AEC and AHEC. **a** Plots of phase angle as a function of frequency for a commercial AEC and AHEC. **b** Nyquist plots of AEC and AHEC. Source data are provided as a Source Data file.

It is informative to compare the electrochemical performances and resistance of AHEC (67 μF) and AEC (22 μF). As shown in Fig. S12a, the AHEC has the comparable phase angle (over 80°) to the AEC at 120 Hz, indicating the fast response of the AHEC at high frequency condition. In addition, the AHEC exhibits a smaller ESR (0.21 Ω cm^2) compared with that (4.1 Ω cm^2) of AEC. This could also be confirmed by the RC time. The AEC possess a smaller RC time of 0.09 ms at 120 Hz than that (0.18 ms) of AHEC. But the capacitance of AHEC is three times higher than that of AEC. Thus, the resistance of the AEC is at least six times than that of AHEC.

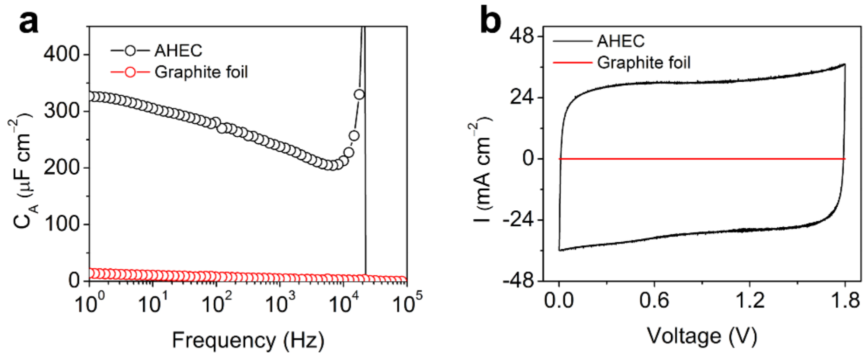

Supplementary Figure 13. Comparison electrochemical performances of AHEC and bare graphite foil. **a** Plots of specific areal capacitance as a function of frequency **b** CV curves for the bare graphite foils-based EC *v.s.* AHEC. Source data are provided as a Source Data file.

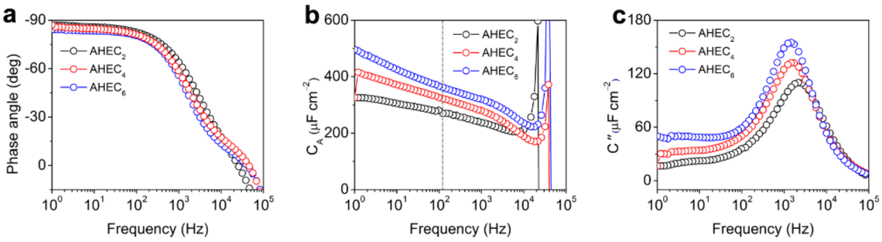

Supplementary Figure 14. Electrochemical Performances of $AHEC_2$, $AHEC_4$, and $AHEC_6$. **a** Plots of phase angle versus frequency. **b** Plots of C_A versus frequency. **c** Plots of imaginary part of specific capacitance versus frequency. Source data are provided as a Source Data file.

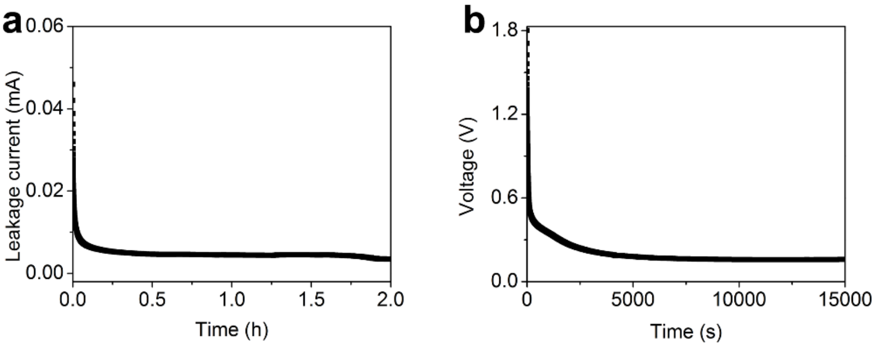

Supplementary Figure 15. Leakage current and self-discharging behaviors of AHEC. **a** Leakage current curve of the AHEC charged at 0.04 mA to 1.8 V and kept at 1.8 V for 2h. **b** Self-discharge curve of the AHEC after charged at 1.8 V for 10 min. Source data are provided as a Source Data file.

As shown in the Supplementary Figure S15, the leakage current of the AHEC is as low as 3.5 μA, indicating the good stability of the AHEC. Meanwhile, the self-discharge curve of AHEC shows a quick decrease in the beginning time. It may be due to the easy adsorption/desorption ion within large porous structure. And then the AHEC can maintain a voltage of 0.16 V after 15000 s.

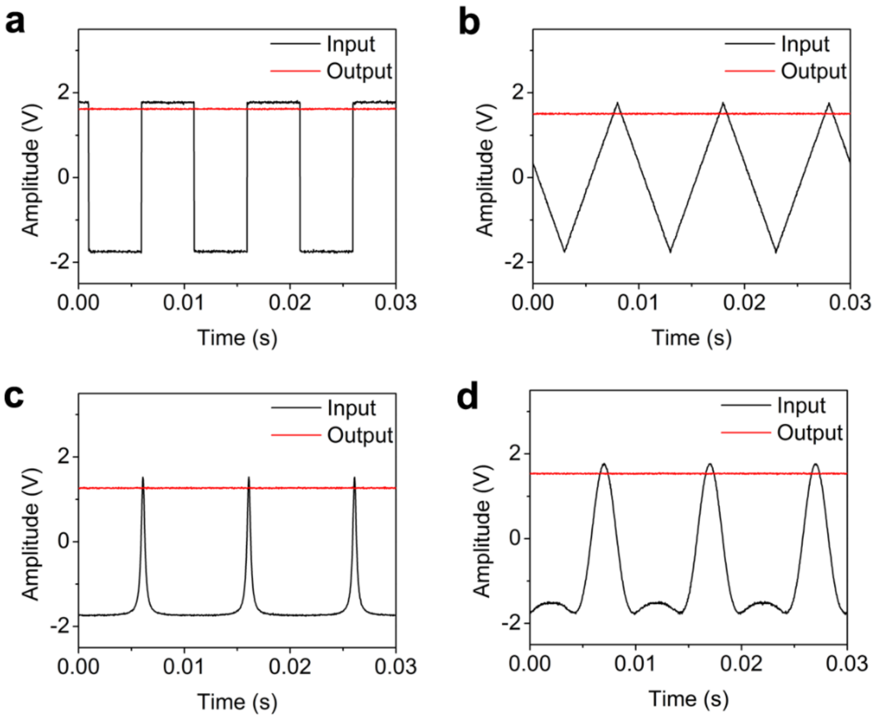

Supplementary Figure 16. Curves line-filtering performance of the AHEC unit. Square **a**, triangle **b**, Lorentz **c**, and hill **d** waveforms. Source data are provided as a Source Data file.

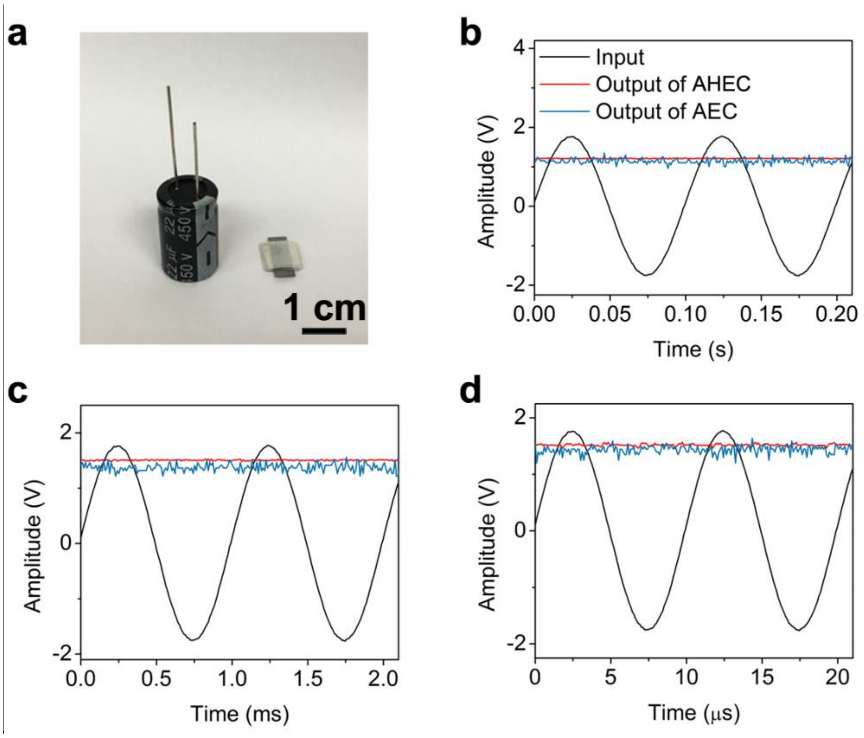

Supplementary Figure 17. AC line-filtering performance of the AHEC unit at different frequencies. **a** Optical images of the AHEC unit and an AEC (22 μF/ 450V). **b** 10 Hz, **c** 1,000 Hz, and **d** 10,000 Hz. Source data are provided as a Source Data file.

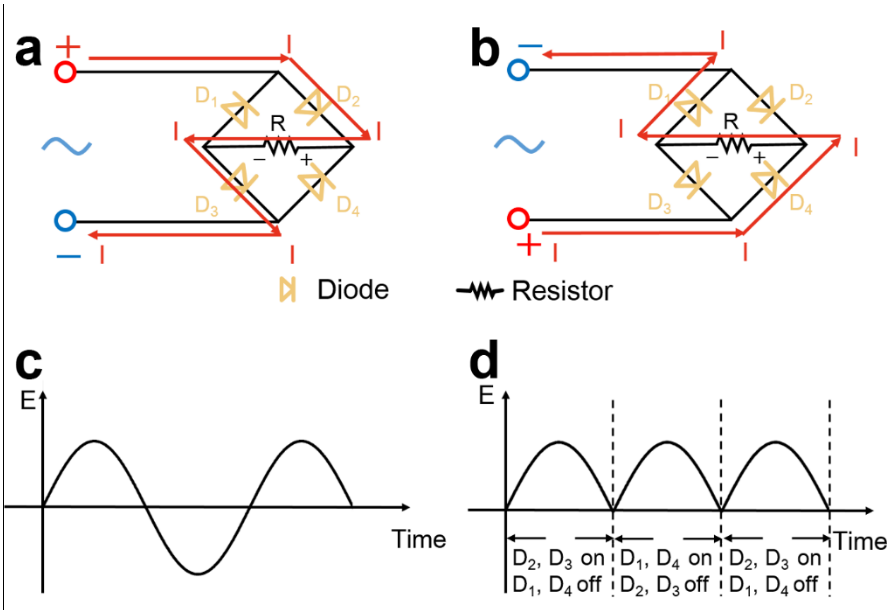

Supplementary Figure 18. The rectification mechanism of the bridge rectifier.

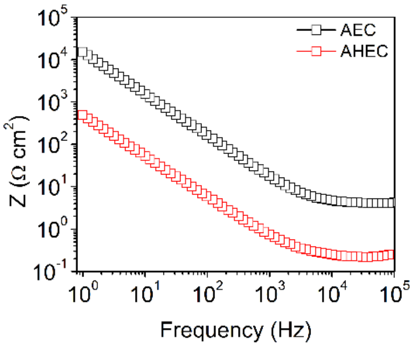

Supplementary Figure 19. Plots of total impedance of AEC and AHEC at frequency of 1 Hz to 100,000 Hz. Source data are provided as a Source Data file.

Supplementary Figure S19 shows the impedance of AEC and AHEC within the frequency range from 1 Hz to 100,000 Hz. Both the AEC and AHEC exhibits a typical properties of RCL circuit including the capacitive impedance ($1/2\pi fC$), inductive impedance ($2\pi fL$), and ESR. The ESR could be read at the frequency that $1/2\pi fC = 2\pi fL$. It is also the lowest point in the curves. And at this point, the corresponding frequency is known as self-resonant frequency (SRF). For the two-terminal electrical component, the SRF is the separation determining that the component behaves like a capacitor or an inductor. In our AHEC and the AEC, the SRF is approximately 20 kHz. Thus, below the 20 kHz, they can be used as the filtering capacitor. The smaller ESR ($0.21\Omega\ cm^2$) and large capacitance of the AHEC determines it possess low impedance within the workable frequency range.

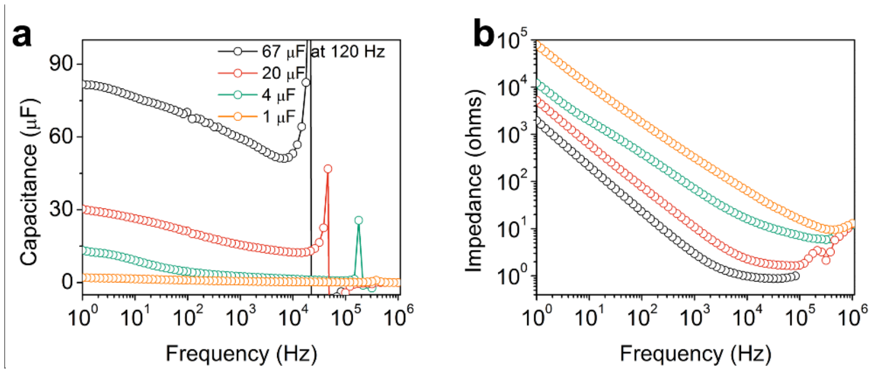

Supplementary Figure 20. Plots of capacitances and impedances versus frequency of AHECs with different mass loading: **a** capacitances; **b** impedances. Source data are provided as a Source Data file.

According to the equation of SRF ($f_0 = 1/2\pi\sqrt{LC}$), reducing the inductance and capacitance could increase the SRF. Generally, the inductance result from the lead of the circuit. The capacitance relies on the mass loading of electrode materials. We could prepare a series of AHECs with different SRFs of by adjusting their capacitances. As shown in Supplementary Figure S20, with decreasing the capacitances in the sequence: 67 μF, 20 μF, 4 μF, and 1 μF, the SRF of the AHEC is about 20 kHz, 80 kHz, 200 kHz, and 500 kHz, respectively. These indicate that the workable frequency of the AHEC could be broadened by changing the capacitance of the AHEC. However, the ESR of the AHEC also increases when its capacitance is relatively low, which is undesirable for the low impedance filtering. Thus, adjusting the capacitance is only an alternative method.

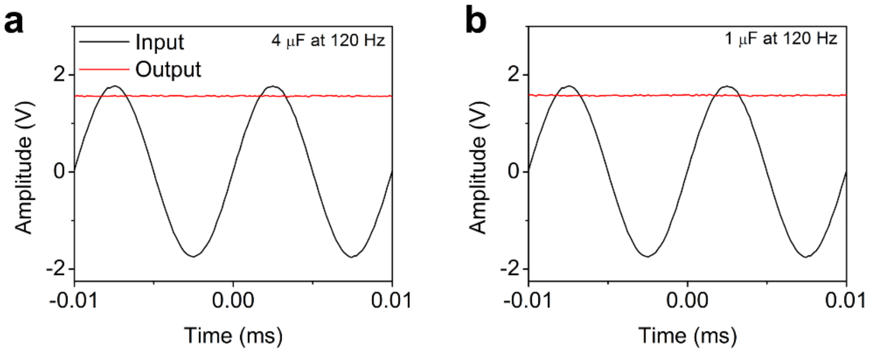

Supplementary Figure 21. AC line-filtering performance of the AHEC unit with different capacitance at 100 kHz: **a** 4 μF at 120 Hz; **b** 1 μF at 120 Hz. Source data are provided as a Source Data file.

Furthermore, in order to check the increasing SRF, the AHECs with different capacitances of 4 μF and 1 μF at 120 Hz were performed on the AC line-filtering experiments. As shown in Supplementary Figure S21, both of them show a good filtering performance at 100 kHz, indicating adjusting the capacitance of the AHEC could be an alternative method to broaden its available frequency range.

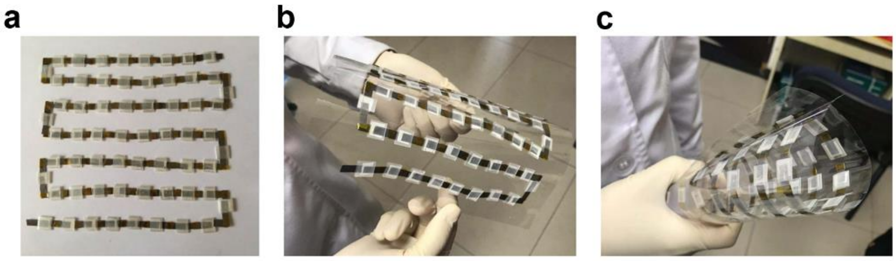

Supplementary Figure 22. A paper-like AC-line filtering device was produced that can be bent and curved.

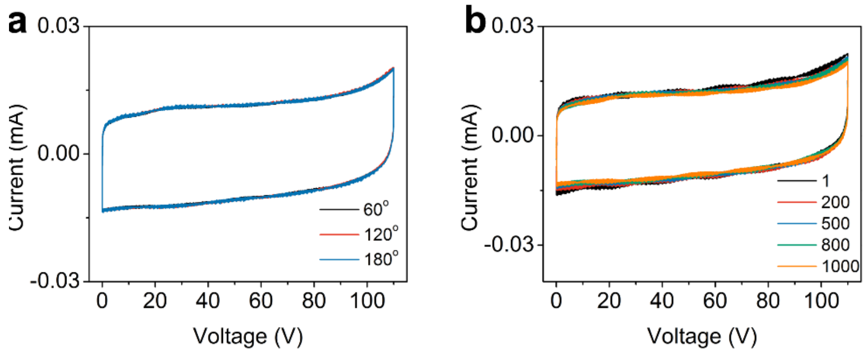

Supplementary Figure 23. Flexibility and mechanical stability of integrated AHECs at work condition. **a** CV curves of integrated AHECs in at different bending angles. **b** CV curves of integrated AHECs after repeated bending cycles at 90°. Source data are provided as a Source Data file.

The integrated AHECs also possess good flexibility and mechanical stability. When the integrated AHECs were bent from 60° to 180°, its CV curves showed a negligible distortion (Supplementary Figure 23a). Meanwhile, it also exhibits a good working condition after 1000 bending cycles at a bend angle of 90°, which can be observed by the similar CV curves of the integrated AHECs (Supplementary Figure 23b).

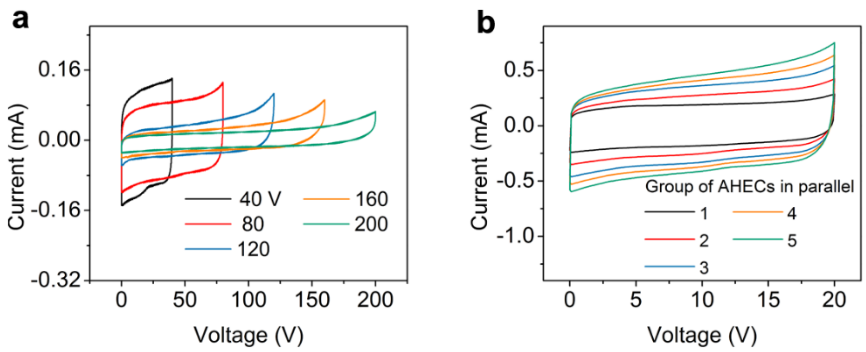

Supplementary Figure 24. The electrochemical performances of AHECs connected in series and in parallel. **a** CV curves of AHECs connected in series. **b** CV curves of AHECs connected in parallel, one group of AHECs was composed of 14 AHEC units connected in series. Source data are provided as a Source Data file.

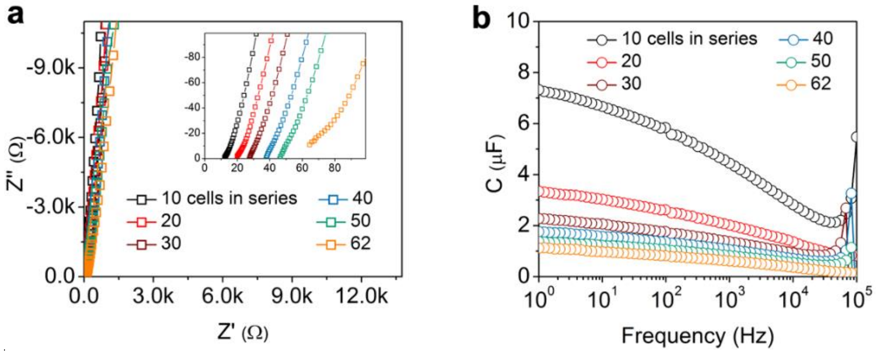

Supplementary Figure 25. EIS characterizations of AEHCs connected in series. **a** Nyquist plots of the AHECs connected in series of different number. **b** Capacitances of the AHECs connected in series of different number. Source data are provided as a Source Data file.

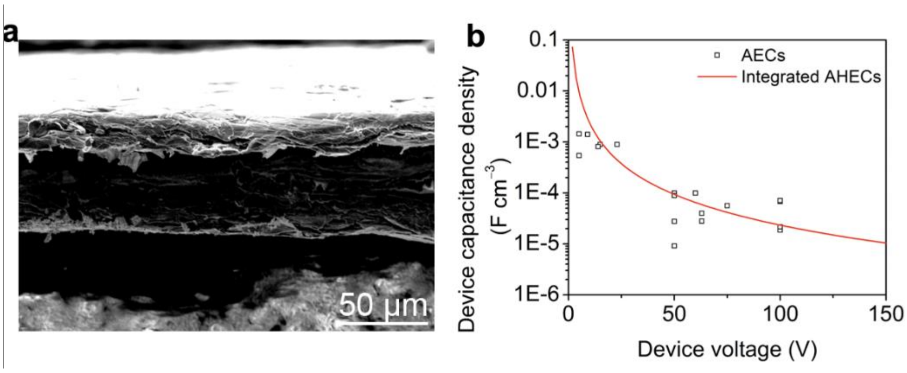

Supplementary Figure 26. Volumetric capacitance comparison of integrated AHECs and commercial AECs. **a** The thickness of an AHEC including current collector, electrode materials, and separator. **b** The volumetric capacitance variation as function of the voltages of the integrated AHECs and AECs. The data of the AECs are collected from the previous literature and local stores.[7] Source data are provided as a Source Data file.

Comparing the volumetric capacitance of AHEC to that of AECs used in line-filtering is informative.[7] The operating voltage can be increased by connecting the AHEC units in series. The thickness of the planner designed AHECs (Fig. 5d) was about 60 μm (current collector, electrode materials, cellulose separator, and uncompacted gaps), which is actually observed by the cross-section SEM image of an AHEC unit (Supplementary Figure S26a). With the C_A of 362 μF cm^{-2} for the AHEC$_6$, the C_v of one unit is calculated to 0.06 F cm^{-3}. Assuming the C_v variation of integrated AHECs is only relative to the amounts of the AHEC units without any other loss. As shown in Supplementary Figure S26b, the integrated AHECs have a higher volumetric capacitance (C_v) than AECs up to 16 V, and was comparable to AECs up to 100 V.

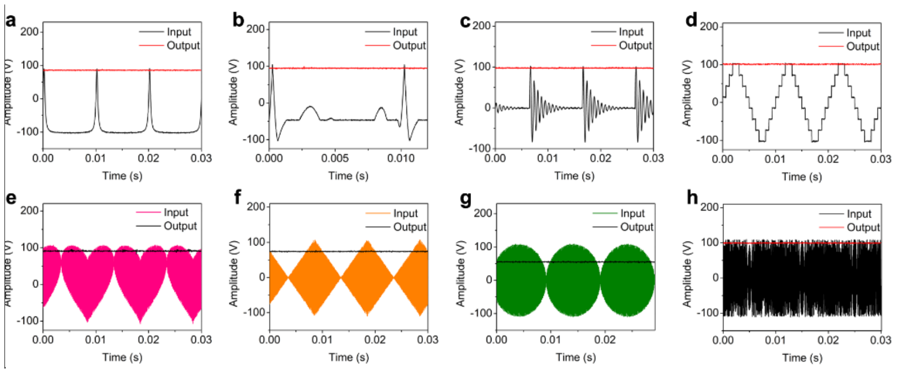

Supplementary Figure 27. The filtering performances of the connected AHECs in series under 110 V for arbitrary waveforms. The input signals are Lorentz **a**, electrocardiogram **b**, decaying **c**, stair **d**, heart **e**, rhombus **f**, ellipse **g**, and violent noise waveforms **h**. Source data are provided as a Source Data file.

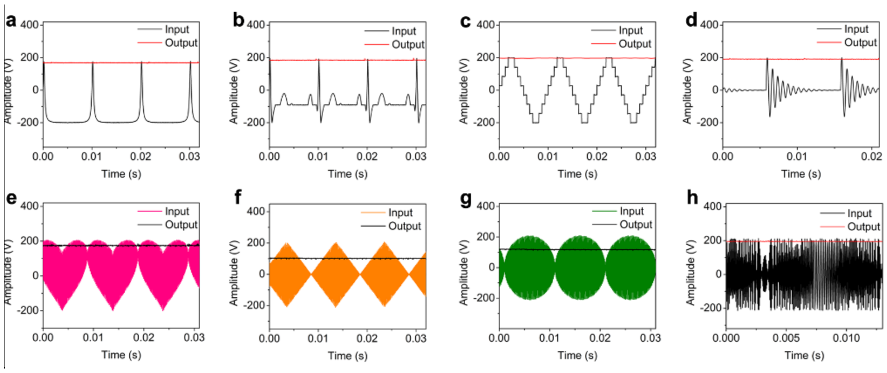

Supplementary Figure 28. The filtering performances of the connected AHECs in series under 200 V for arbitrary waveforms. The input signals are Lorentz **a**, electrocardiogram **b**, stair **c**, decaying **d**, heart **e**, rhombus **f**, ellipse **g**, and violent noise waveforms **h**. Source data are provided as a Source Data file.

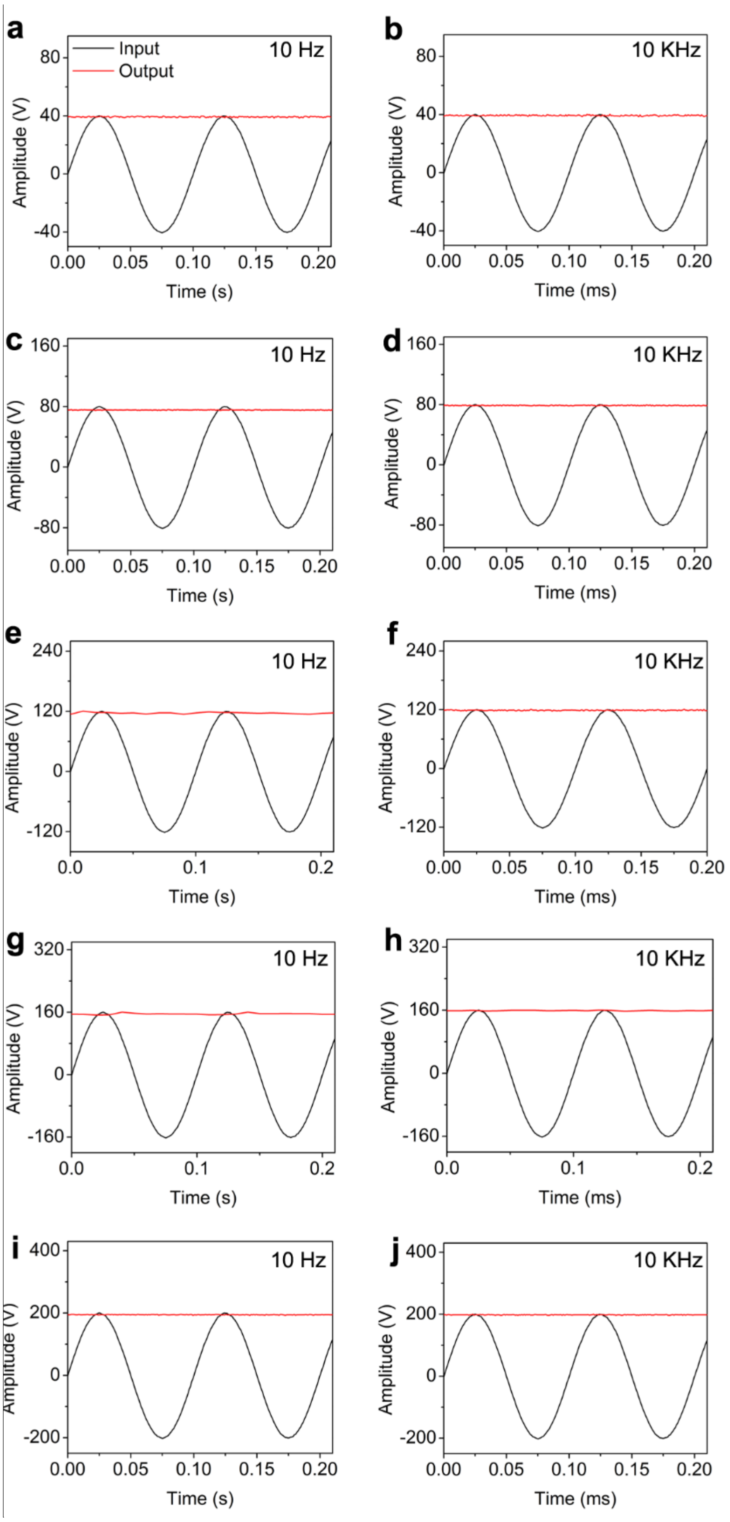

Supplementary Figure 29. The filtering performances of the connected AHECs in series (40 V to 200 V) at different frequency. 40 V: 10 Hz **a** and 10 KHz **b**. 80 V: 10 Hz **c** and 10 KHz **d**. 120 V: 10 Hz **e** and 10 KHz **f**. 160 V: 10 Hz **g** and 10 KHz **h**. 200 V: 10 Hz **i** and 10 KHz **j**. Source data are provided as a Source Data file.

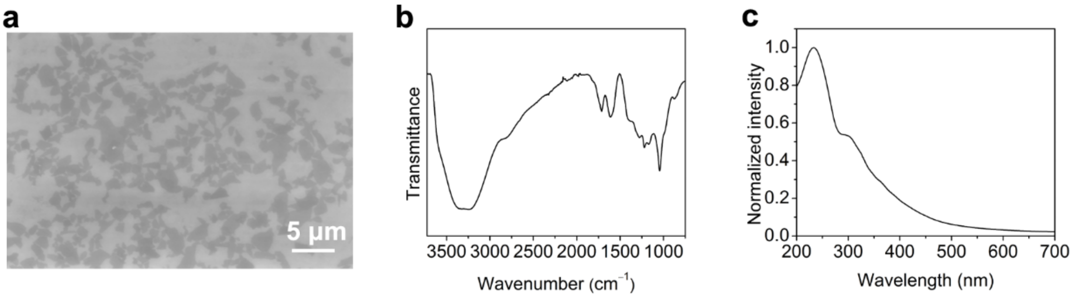

Supplementary Figure 30. Characterizations of GO sheets. **a** SEM images of GO sheets with an average lateral size of 2-4 μm. **b** Attenuated total reflection Fourier transform infrared (FTIR) spectra of GO. **c** UV-vis spectra of GO. Source data are provided as a Source Data file.

For clarity, physical and chemical information of the GO were given by Supplementary Figure S30. A relatively small size of 2-4 μm for GO sheets could be observed by the SEM images, which is advantageous to uniform growth of ErGO during electrochemcial deposition process. ATR-FTIR spectral studies show the oxygen groups located on th GO sheets including C=O (1740−1720 cm^{-1}), C−O−C (~1000 cm^{-1}), C−O, (1230 cm^{-1}), and −OH (3600−3300 cm^{-1}), which are also confirmed by XPS analysis. And UV-vis adsorption spectrum of GO exhibits a main adsorption peak at around 230 nm from the π–π^* transition of conjugated ketones or dienes. The wide adsorption region between 270 and 600 nm belongs to the conjugated aromatic domains.

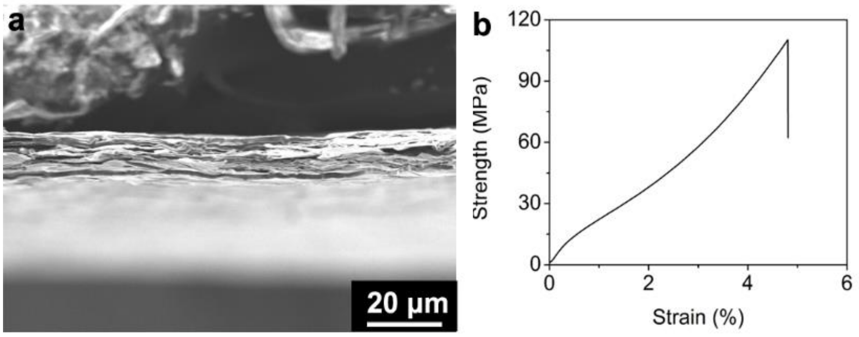

Supplementary Figure 31. Mechanical properties of graphite foil. **a** Cross-section SEM image of graphite foil. **b** Typical stress-strain curve of graphite foil. Source data are provided as a Source Data file.

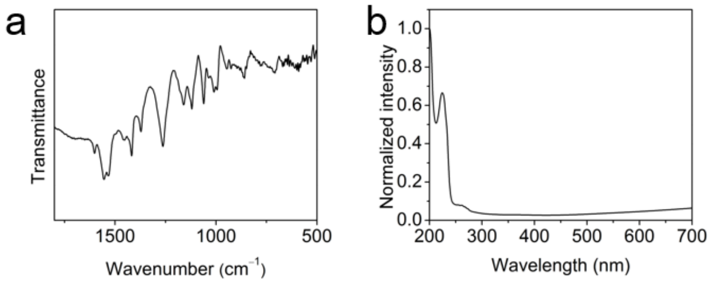

Supplementary Figure 32. Characterizations of PH1000. **a** FTIR spectra of PH 1000. **b** UV-vis spectra of PH 1000. Source data are provided as a Source Data file.

Supplementary Figure S32. Shows the basic chemical structures of the PH1000, which is the precursor of PEDOT positive electrode. The ATR-FTIR spectra provide more detailed information of the stretching vibrations of benzene sulfonate (1172, 1128, 1035, and 1005 cm^{-1}), indicating the existence of PSS. In addition, in the UV-vis spectra, the higher energy peak at ~340 nm is assigned to the n–π* transition in the PEDOT backbone and the broad band above 600 nm corresponds to bipolaron subgap states of the sample.

Supplementary Table 1. Comparisons of the electrochemical performance parameters of various aqueous ECs for AC-line filtering.

Electrodes	E_A [μF V^2 cm^{-2}] at 120 Hz	−Phase angle [°] at 120 Hz	C_A [μF cm^{-2}] at 120 Hz	τ_0 [ms]	τ_{RC} [ms]	Voltage window [V]	References
$AHEC_2$	438	82.0	270	0.464	0.184	1.8	This work
$AHEC_4$	524	80.4	323	0.597	0.222	1.8	This work
$AHEC_6$	586	79.8	362	0.679	0.235	1.8	This work
VOGNs[a]	44	82	87.5	0.067	0.200	1.0	8
CB[b]	280	75	559	1.560	0.354	1.0	9
ErGO[c]	91	84.0	283	0.238	1.350	0.8	10
VOG[d]	146	82	360	0.248	0.205	0.9	11
SWNT[e]	192	81	601	0.702	0.2	0.8	12
NHG[f]	153	83.6	478	0.455	0.146	0.8	13
EOG/CCP[g]	122	83	300	0.083		0.9	14
UEC[h]	318	83.6	994	0.588	0.15	0.8	15
EOG/CNF[i]	118	81.5	370	0.070		0.8	16
UDLCs[j]	95	85	190	0.250	0.212	1	17
GMF[k]	98	82.3	306		0.32	0.8	18
MPHM[l]	179	79.1	560	0.71	0.29	0.8	19

[a] VOGNs = Vertically oriented graphene nanosheets;
[b] CB = the carbon black;
[c] ErGO = Electrochemically reduced graphene oxide;
[d] VOG = Vertically oriented graphene;
[e] SWNT = single walled multi-walled carbon nanotube film;
[f] NHG = nitrogen-doped holey graphene;
[g] EOG/CCP= edge oriented multilayer graphene/thin-graphite in carbonized cellulous paper;
[h] UEC = ultrahigh-rate electrochemical capacitors;
SWNT = single walled multi-walled carbon nanotube film;
[i] EOG/CNF = 3D edge-oriented graphene (EOG) was grown encircling carbon nanofiber (CNF);
[j] UDLCs = unzipped structure-based electrode;
[k] GMF = graphene nanomesh film;
[l] MPHM = MXene/PEDOT:PSS hybrid materials

Supplementary Table 2. Comparisons of the AC-line filtering performance parameters of various ECs with AC-line filtering tests.

ECs	Frequency range [Hz]	Highest voltage window [V]	References
AHEC	1~10000	110	This work
VOGNs[a]	50000	2.5	8
CB[b]	30000	1	9
IEDC[c]	50	100	20
SSFEC[d]	60	4.3	21
GOMC/CNT[e]	60	3.6	16
CBC[f]	60	4.2	22
OEC[g]	60	2.5	23
KBEC[h]	60	4.7	24
GPEC[i]	60	1	25
EOG/CN[j]	60	3	17
MPHM[k]	60~10000	0.8	19

[a] VOGNs = Vertically oriented graphene nanosheets;
[b] CB = the carbon black;
[c] IEDC = integrated energy device circuits (interdigital single-walled carbon nanotube electrode);
[d] SSFEC = solid-state flexible electrochemical capacitor;
[e] GOMC/CNT = graphic ordered mesoporous carbon and carbon nanotubes;
[f] CBC = carbonized bacterial cellulose aerogel;
[g] OECs = organic ECs with deeply electrochemically reduced graphene;
[h] KBEC = Ketien black electrochemical capacitor;
[i] GPECs = ECs based on graphene/PEDOT:PSS composite film;
[j] EOG/CN = edge-oriented graphene on carbon nanofiber;
[k] MPHM = MXene/PEDOT:PSS hybrid materials

S3. Supplementary Referances

1. Yao, B. et al. Ultrahigh-conductivity polymer hydrogels with arbitrary structures. *Adv. Mater.* **29**, 1700974 (2017).
2. Cruz-Cruz, I., Reyes-Reyes, M. & Lopez-Sandoval, R. Formation of polystyrene sulfonic acid surface structures on poly(3,4-ethylenedioxythiophene): Poly(styrenesulfonate) thin films and the enhancement of its conductivity by using sulfuric acid. *Thin Solid Films* **531**, 385−390 (2013).
3. Kim, N. et al. Highly conductive PEDOT: PSS nanofibrils induced by solution-processed crystallization. *Adv. Mater.* **26**, 2268−2272 (2014).
4. Ouyang, J. et al. On the mechanism of conductivity enhancement in poly (3,4-ethylenedioxythiophene): poly(styrene sulfonate) film through solvent treatment. *Polymer* **45**, 8443−8450 (2004).
5. McCarthy, J. E., Hanley, C. A., Brennan, L. J., Lambertini, V. G. & Gun'ko, Y. K. Fabrication of highly transparent and conducting PEDOT:PSS films using a formic acid treatment. *J. Mater. Chem. C* **2**, 764−770 (2014).
6. Chen, J. et al. Water-enhanced oxidation of graphite to graphene oxide with controlled species of oxygenated groups. *Chem. Sci.* **7**, 1874-1881 (2016).
7. Miller. J. R., Outlaw R. A. Vertically-oriented graphene electric double layer capacitor designs batteries and energy storage. *J. Electrochem. Soc.* **162**, A5077−A5082 (2015)
8. Miller, J. R., Outlaw, R. A. & Holloway, B. C. Graphene double-layer capacitor with ac line-filtering performance. *Science* **329**, 1637−1639 (2010).
9. Kossyrev, P. Carbon black supercapacitors employing thin electrodes. *J. Power Sources* **201**, 347−352 (2012).
10. Sheng, K., Sun, Y., Li, C., Yuan, W. & Shi, G. Ultrahigh-rate supercapacitors based on eletrochemically reduced graphene oxide for ac line-filtering. *Sci. Rep.* **2**, 247 (2012).
11. Ren, G., Pan, X., Bayne, S. & Fan, Z. Kilohertz ultrafast electrochemical supercapacitors based on perpendicularly-oriented graphene grown inside of nickel foam. *Carbon* **71**, 94−101 (2014).
12. Rangom, Y., Tang, X. S. & Nazar, L. F. Carbon nanotube-based supercapacitors with excellent ac Line filtering and rate capability via improved interfacial impedance. *ACS Nano* **9**, 7248−7255 (2015).
13. Zhou, Q., Zhang, M., Chen, J., Hong, J. D. & Shi, G. Nitrogen-doped holey graphene film-based ultrafast electrochemical capacitors. *ACS Appl. Mater. Inter.* **8**, 20741−20747 (2016).
14. Ren, G., Li, S., Fan, Z.-X., Hoque, M. N. F. & Fan, Z. Ultrahigh-rate supercapacitors with large capacitance based on edge oriented graphene coated carbonized cellulous paper as flexible freestanding electrodes. *J. Power Sources* **325**, 152−160 (2016).
15. Zhang, M. et al. An ultrahigh-rate electrochemical capacitor based on solution-processed highly conductive PEDOT:PSS films for AC line-filtering. *Energy Environ. Sci.* **9**, 2005−2010 (2016).

16. Yoo, Y., Kim, M.-S., Kim, J.-K., Kim, Y. S. & Kim, W. Fast-response supercapacitors with graphitic ordered mesoporous carbons and carbon nanotubes for AC line filtering. *J. Mater. Chem. A* **4**, 5062−5068 (2016).
17. Islam, N., Warzywoda, J. & Fan, Z. Edge-oriented graphene on carbon nanofiber for high-frequency supercapacitors. *Nano-Micro Lett.* **10**, 9 (2018).
18. Zhang Z. et al. Scalable fabrication of ultrathin free-standing graphene nanomesh films for flexible ultrafast electrochemical capacitors with AC line-filtering performance. *Nano Energy* **50**, 182−191 (2018).
19. Gund, G. S. et al. MXene/polymer hybrid materials for flexible AC-filtering electrochemical capacitors. *Joule* **3**, 164−176 (2019).
20. Laszczyk, K. U. et al. Lithographically integrated microsupercapacitors for compact, high performance, and designable energy circuits. *Adv. Energy Mater.* **5**, 1500741 (2015).
21. Kang, Y. J., Yoo, Y. & Kim, W. 3-V solid-state flexible supercapacitors with ionic-liquid-based polymer gel electrolyte for AC line filtering. *ACS Appl. Mater. Inter.* **8**, 13909−13917 (2016).
22. Islam N. et al. High-frequency electrochemical capacitors based on plasma pyrolyzed bacterial cellulose aerogel for current ripple filtering and pulse energy storage. *Nano Energy* **40**, 107−114 (2017).
23. Chi, F. et al. Graphene-based organic electrochemical capacitors for AC line filtering. *Adv. Energy Mater.* **7**, 1700591 (2017).
24. Yoo, Y., Park, J., Kim, M.-S. & Kim, W. Development of 2.8 V Ketjen black supercapacitors with high rate capabilities for AC line filtering. *J. Power Sources* **360**, 383−390 (2017).
25. Zhang, M. et al. Robust graphene composite films for multifunctional electrochemical capacitors with an ultrawide range of areal mass loading toward high-rate frequency response and ultrahigh specific capacitance. *Energy Environ. Sci.* **11**, 559−565 (2018).
